# Supplementary material for: Chromothripsis during telomere crisis is independent of NHEJ, and consistent with a replicative origin
Source: Genome Res. 2019 May;29(5):737–49. doi: 10.1101/gr.240705.118 (PMC6499312; doi:10.1101/gr.240705.118)
Supplement: Supplemental Material [file supp_gr.240705.118_Supplemental_file_1.zip › contigs/annotated_contigs/DB79/contig.2.DB79_length_475_mean_cov_21.0315789474.docx]

**DB79_length_475_mean_cov_21.0315789474**

```
TAGAGATGGATGGTGGTGATGTTGCACAACAATGTGAATGTACTTAATGCCACTGAACTGCACACTTAAAAGGCTAAGATAGTGAATTT
 >chr2:7697454-7697708 + E=2e-141
TATGTTAGGTATATTTTACCAAAATTTAGAACTTTTAAAAAGGAAAATATCATCTACTGGTCTTTAGAGTACAGACTTCAGAAGCCCAA

CATAAAATCATGCAGACCTGTAGGAAGCTGTTGCCTATGTCCAGCCCCAGACATGATAGTAGCTTGCACTATTA|GT|ATCGTTTGGCC
                                                                           >chr2:741635
TGGAAGCATTTCAAATTAGGAAGTGGGCTATTAAGGGAAATAGTTGTTCCTACTAATGCCACGTAGACAAGTTCTATGGCAGGGCTTTG
7-7416580 + E=1e-122
CGCTAGCAATCACAGGTAAAATGACACGAGTGTATTATTTTAGCTAGCACAGATTGTAGACAGATTCTCCCTAATATGTTAGAAAGGCC

TCCTTATAATCTCATGATAGAATCATTCACTT
```
